# A Dental Surgery in a Factory

**Published:** 1914-01-31

**Authors:** 


### A Dental Surgery in a Factory.

Realising that much of the ill-health of those employed in factories is due to bad condition or loss of teeth, and further with the object of protecting their employees from unqualified practitioners, the directors of a Bristol firm (W. D. and H. O. Wills) have established a dental surgery in their factory. A qualified dental surgeon is in charge, assisted by a nurse, and every opportunity is given for the work to be carried out in the most efficient manner possible.

The department consists of a well-lighted surgery, waiting and recovery rooms, and a mechanical laboratory for the manufacture of artificial dentures. Treatment is free during working hours for all members of the staff and employees; the only condition imposed is that the advice of the dental surgeon shall be accepted. Ordinary extractions and fillings are free, but where a filling ot gold is required the cost of the gold has to be borne by the patient; and if gas is needed for extraction a charge of 6d. is made. All artificial plates are supplied at cost price.

All candidates for employment in the factory are required to pass the dentist, and no one is permitted to commence work until the requirements prescribed have been carried out. The employees undergo a half-yearly examination, records of which are carefully preserved. The following figures supplied through the kindness of the firm give some idea of the work carried on during the last twelve months:

| | |
|---|---|
| Number of visits by employees ... ... | 6,490 |
| Extractions (ordinary) ... ... ... | 1,691 |
| Extractions (gas) ... ... ... ... | 2,760 |
| Gas administrations ... ... ... ... | 800 |
| Fillings ... ... ... ... ... ... | 518 |
| Fillings (gold) ... ... ... ... ... | 6 |
| Scalings ... ... ... ... ... | 60 |
| Crowns ... ... ... ... ... ... | 10 |
| Artificial plates ... ... ... ... | 288 |

Tooth-brushes are supplied at 2d. each and tooth-powder at 1d. per tin. The sale of these has amounted to about 2,000 brushes and 5,000 tins of powder.

A great difficulty has been encountered in persuading the younger employees to have teeth filled and not extracted—another comment upon the influence of the unqualified practitioner, who for obvious reasons prefers the extraction to the filling—but the prejudice is being overcome, and the dental surgeon reports that there is an increasing number of those who wish for the conservative work.

